# Comparison of Significant Carotid Stenosis for Nasopharyngeal Carcinoma between Intensity-Modulated Radiotherapy and Conventional Two-Dimensional Radiotherapy

**DOI:** 10.1038/s41598-018-32398-y

**Published:** 2018-09-17

**Authors:** Wang Liao, Haihong Zhou, Shengnuo Fan, Yuqiu Zheng, Bei Zhang, Zhongyan Zhao, Songhua Xiao, Shoumin Bai, Jun Liu

**Affiliations:** 1 Department of Neurology, Sun Yat-sen Memorial Hospital, Sun Yat-sen University, Guangzhou, 510120 China; 2 Department of Psychiatry, McLean Hospital, Harvard Medical School, Belmont, MA 02478 USA; 3 Department of Neurology, Affiliated Hospital of Guangdong Medical University, Zhanjiang, 524000 China; 4 Department of Neurology, the First Affiliated Hospital of Guangdong Pharmaceutical University, Guangzhou, 510080 China; 5 Department of Neurology, People’s Hospital of Hainan Province, Haikou, 570311 China; 6 Department of Oncology, Sun Yat-sen Memorial Hospital, Sun Yat-sen University, Guangzhou, 510120 China; 7 Guangdong Province Key Laboratory of Brain Function and Disease, Zhongshan School of Medicine, Sun Yat-sen University, Guangzhou, Guangdong 510120 China; 8 Laboratory of RNA and Major Diseases of Brain and Heart, Sun Yat-sen Memorial Hospital, Sun Yat-sen University, Guangzhou, 510120 China

## Abstract

Radiotherapy (RT) serves as the most efficient treatment for nasopharyngeal carcinoma (NPC) and can cause carotid stenosis. This work compared the incidence of significant carotid stenosis between intensity-modulated radiotherapy (IMRT) and two-dimensional conventional radiotherapy (2D-RT) for NPC and explored the risk factors. We retrospectively reviewed 233 cases with NPC who underwent carotid ultrasound post IMRT or 2D-RT from 2006 to 2015. The incidence of significant stenosis after RT was 19.3%. Significant stenosis was identified in 20 (14.6%) of 137 patients treated with IMRT and 25 (26.0%) of 96 patients with 2D-RT, respectively (*p* = 0.035). Multivariate logistic analysis indicated age (odds ratio = 1.054, 95% CI = 1.011–1.099, *p* = 0.014), radiation technique (IMRT) (odds ratio = 0.471, 95%CI = 0.241–0.919, *p* = 0.027) and time interval (odds ratio = 1.068, 95%CI = 1.033–1.105, *p* = 0.001) as independent predictors for significant carotid stenosis. Our study suggests that IMRT was associated with decreased incidence of significant carotid stenosis versus 2D-RT for NPC. Prevention and carotid ultrasound should be considered for older NPC survivors with longer interval from RT, especially those treated with 2D-RT.

## Introduction

Nasopharyngeal carcinoma (NPC), a rare disease in the world, is among the most common causes of head and neck cancer in southern China, with a rate of 15–50 per 100,000 people^1,2^. Because NPC is highly radiosensitive, radiotherapy (RT) serves as the most efficient treatment, with the 5-year survival rate above 50%^3^. However, late complications, such as those reported in our previous work (optic neuropathy, brachial plexus injury and brain necrosis)^4–6^, have shown an increasing problem for RT treatment of NPC. While commonly used to prevent nodal metastasis, RT contributes to atherosclerosis of the irradiated vessels and increases the risk of vascular stenosis, which may lead to transient ischemic attacks (TIA) or ischemic stroke^7^.

Intensity-modulated RT (IMRT) is an innovative technology for optimizing the radiation dose distribution and extricating normal tissues from radiation-induced injury^8^. In contrast to conventional two-dimensional RT (2D-RT), IMRT has been reported to have decreased risk of temporal lobe injury^9^ and mastoiditis^10^. Nonetheless, the influence of IMRT on carotid stenosis remains unknown, nor is there a routine to screen patients post RT for carotid stenosis during follow up^11^. Therefore, to investigate the incidence of carotid artery stenosis (≥50%) and the risk factors with IMRT, we retrospectively analyzed 233 cases of NPC treated with IMRT, and compared with those treated with 2D-RT using Doppler ultrasound. Our study implied that IMRT reduced the incidence of significant carotid stenosis, and that prevention and carotid ultrasound should be considered for older NPC survivors.

## Results

### Patient characteristics

Baseline demographic and clinical information of the 233 cases included in this study is displayed in Table 1. The mean age at RT was 51.4 ± 8.4 years old. The patients included 186 males and 47 females. 5.6% had stage I disease, 20.2% had stage II, 53.6% had stage III, and 20.6% had stage IV (Table 1).

**Table 1:** Baseline characteristics of patient with NPC.

Parameter	All Patients (%)	2D-RT (%)	IMRT (%)	*p*-Value
	n = 233	n = 96	n = 137	
Age median (range) (years)	51 (24–75)	51 (29–75)	52 (24–72)	0.929
Gender				0.228
Male	186 (79.8)	73 (76.0)	113 (82.5)	
Female	47 (20.2)	23 (24.0)	24 (17.5)	
Stages group^a^				0.430
I	13 (5.6)	3 (3.1)	10 (7.3)	
II	47 (20.2)	20 (20.8)	27 (19.7)	
III	125 (53.6)	50 (52.1)	75 (54.7)	
IVA-B	48 (20.6)	23 (24.0)	25 (18.2)	
Chemotherapy				0.052
Yes	162 (69.5)	60 (62.5)	102 (74.5)	
No	71 (30.5	36 (37.5)	35 (25.5)	
Smoking				0.294
Yes	64 (27.5)	30 (31.3)	34 (24.8)	
No	169 (72.5)	66 (68.8)	103 (75.2)	
Diabetes				0.383
Yes	31 (13.3)	15 (15.6)	16 (11.7)	
No	202 (86.7)	81 (84.4)	121 (88.3)	
Hypertension				0.054
Yes	85 (36.5)	42 (43.8)	43 (31.4)	
No	148 (63.5)	54 (56.3)	94 (68.6)	
Vascular disease				0.120
Yes	21 (11.3)	12 (16.4)	9 (6.6)	
No	212 (91.0)	84 (87.5)	128 (93.4)	
Atrial fibrillation				0.242
Yes	12 (5.2)	3 (3.1)	9 (6.6)	
No	221 (94.8)	93 (96.9)	128 (93.4)	
Hyperlipidemia				0.850
Yes	28 (12.0)	12 (12.5)	16 (11.7)	
No	205 (88.0)	84 (87.5)	121 (88.3)	

a: Based on the diagnosis by oncologist.

Radiation was delivered using conventional 2-dimensional technique (41.2%) or IMRT (58.8%) (Table 1). The radiation dose was 66.5 ± 4.7 Gy. 162 patients (69.5%) were treated with adjuvant chemotherapy. Median time of the last carotid ultrasound was 69 months (range: 48 to 102 months) after RT.

### Incidence of carotid stenosis (≥50%) after RT and risk factors

**Figure 1:**
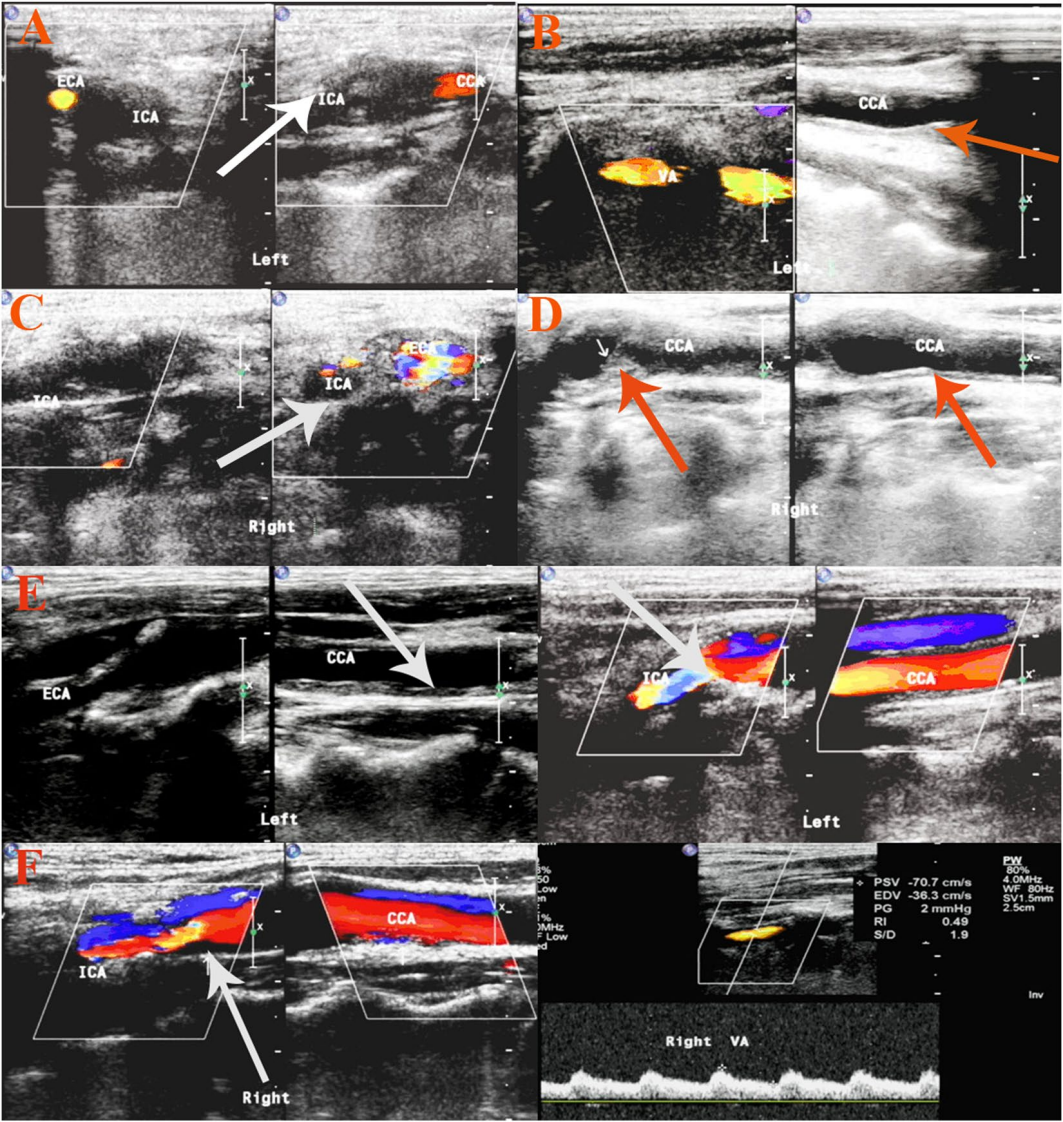
Representative carotid color duplex scan of two patients. (**A**–**D**) was the carotid ultrasonography of one patient. For common carotid artery (CCA) of the right side (R): intimal-medial thickness (IMT) was 1.4 mm; internal carotid artery(ICA): occlusion; For L-CCA: IMT 1.0–1.4 mm, resistance index (RI) 0.74; ICA: occlusion; R- vertebral artery(VA): D 3.9 mm, RI 0.50; L-VA: D 4.4 mm, RI 0.56; (**E**,**F**) is the other. R-CCA: IMT 1.2 mm, RI:0.64; ICA: IMT 1.1 mm, RI: 0.61. external carotid artery (ECA): IMT 1.3 mm, RI 0.67. L-CCA: IMT 2.0 mm, RI 0.8. ICA: IMT 1.3 mm, RI 0.66. R-VA: D 3.4 mm, RI 0.65. L-VA: D 4.0 mm, RI 0.60. Note the carotid artery (stenosis arrowheads).

Significant carotid stenosis (≥50%) was identified in 45 (19.3%) of 233 patients at a median interval of 76 months post RT. A representative carotid ultrasound color duplex scan is displayed in Fig. 1.

Univariate analysis by chi-square test revealed that carotid stenosis was correlated with age (*p* = 0.007), radiation technique (*p* = 0.035), and time interval from radiotherapy (*p* = 0.001). The age in the significant stenosis group (54.1 ± 9.5 years) was older than those in the non-significant stenosis group (50.7 ± 8.0 years) (*p* = 0.015). The time interval from radiotherapy in the significant stenosis group (median, 76, range 59–97 months) was longer versus non-significant stenosis patients (median, 68, range 48–102 months) (*p* < 0.05). There was no significance for the variables of gender (*p* = 0.179), clinical stage (*p* = 0.386), chemotherapy (*p* = 0.236), smoking (*p* = 0.327), diabetes (*p* = 0.325), hypertension (*p* = 0.217), cardio/peripheral vascular disease (*p* = 0.975), atrial fibrillation (*p* = 0.609), and hyperlipidemia (*p* = 0.417) (Table 2).

**Table 2:** Risk factors for significant carotid stenosis in NPC patients after radiotherapy.

Variable	Significant carotid stenosis (%)	Non-Significant carotid stenosis (%)	X^2^	*p*-Value
	n = 45	n = 188		
Gender			1.809	0.179
Male	40 (21.5)	146 (78.5)		
Female	5 (10.6)	42 (89.4)		
Age (years)			7.287	0.007^a^
<50	33 (25.6)	92 (88.5)		
≥50	12 (11.5)	96 (74.4)		
Stages group^a^			3.042	0.386
I	3 (23.1)	10 (76.9)		
II	12 (25.5)	35 (74.5)		
III	19 (15.2)	106 (84.8)		
IVA-B	11 (22.9)	37 (77.1)		
Chemotherapy			1.405	0.236
Yes	28 (17.3)	134 (82.7)		
No	17 (23.9)	54 (76.1)		
Smoking			0.964	0.327
Yes	15 (23.4)	139 (82.2)		
No	30 (17.8)	23 (74.2)		
Diabetes			0.968	0.325
Yes	8 (25.8)	23 (74.2)		
No	37 (18.3)	165 (81.7)		
Hypertension			1.526	0.217
Yes	20 (23.5)	65 (76.5)		
No	25 (16.9)	123 (83.1)		
Vascular disease			0.002	0.975
Yes	4 (19.0)	17 (81.0)		
No	41 (19.3)	171 (80.7)		
Atrial fibrillation			0.263	0.609
Yes	3 (25.0)	9 (75.0)		
No	42 (19.0)	179 (81.0)		
Hyperlipidemia			0.661	0.417
Yes	7 (25.0)	21 (75.0)		
No	38 (18.5)	167 (81.5)		
Radiation technique			4.743	0.03^a^
2D-RT	25 (26.0)	71 (74.0)		
IMRT	20 (14.6)	117 (85.4)		
time interval from radiotherapy (months)			7.371	0.001^a^
<70	15 (33)	105 (55.9)		
≥70	30 (27)	83 (73.5)		

^a^*p* < 0.05.

Table 3 is the endpoint of multivariate logistic regression analysis of those risk factors. It shows that older age at RT (OR = 1.054, 95% CI = 1.011–1.099, *p* = 0.014) and time interval from radiotherapy (OR = 1.068, 95% CI = 1.033–1.105, *p* = 0.001) were associated with higher carotid stenosis (≥50%) risk, whereas radiotherapeutic technique (IMRT) was correlated with a decreased risk (OR = 0.471, 95%CI = 0.241–0.919, *p* = 0.027).

**Table 3:** Result of multivariate logistic regression analysis.

Variable	odds ratio	95% CI	*p* value
Age (years)	1.058	1.013–1.105	0.011
Radiation technique (IMRT)	0.459	0.228–0.926	0.030
Time interval from radiotherapy (months)	1.068	1.033–1.105	0.001

CI = confidence interval.

### Comparison between IMRT and 2D-RT

Demographic information and clinical characteristics of both 2D-RT and IMRT groups are shown in Table 1. There was no significant difference between age, gender, and atherosclerosis hazard factors including age, smoking, hypertension, diabetes, vascular disease, and concurrent chemotherapy. Significant stenosis was identified in 20 of 137 patients treated with IMRT and 25 of 96 patients with 2D-RT, respectively (*p* = 0.035) (Table 2). Stenosis after RT was more commonly seen in 2D-RT (26.0%) versus IMRT (14.6%). However, there was no distinction between time interval of the 2D-RT group (median 77, range 59 to 97 months) and the IMRT group (median 72, range 61 to 93 months) (*p* = 0.204).

Furthermore, the distribution of the stenosis within the carotid was analyzed in terms of common carotid artery (CCA), internal carotid artery (ICA) and carotid bulb. External carotid artery (ECA) was not included since insufficient information was given from most of the ultrasound scan report. We found that the incidence of significant carotid artery, internal carotid artery and carotid bulb stenosis after IMRT (11.0%, 8.8%, 8.1%, respectively) were all lower than 2D-RT (21.9%, 17.8%, 17.8%, respectively) (Table 4).

**Table 4:** Incidence and distribution of carotid stenosis in 2D-RT and IMRT group.

Stenotic artery	2D-RT (%)	IMRT (%)	X^2^	*p*-Value
	n = 96	n = 137		
Common carotid artery	21 (21.9)	15 (11.0)	5.158	0.024
Internal carotid artery	17 (17.8)	12 (8.8)	4.148	0.042
Carotid bulb	17 (17.8)	11 (8.1)	5.002	0.026

### Outcomes

Most of the 45 patients diagnosed with carotid stenosis (≥50%) took oral medicine like lipid-lowering drugs and antiplatelet drugs. Five patients were subjected to operative intervention, 4 patients with carotid endarterectomy and 7 with carotid artery stenting. Among them, 5 patients developed restenosis after 2 years’ follow-up.

## Discussion

The literature has demonstrated that RT might cause carotid stenosis^8,12,13^. As an alternative, IMRT is being widely adopted for treating NPC patients^14,15^. Here we showed that 14.6% of the patients treated with IMRT developed carotid stenosis, whereas the incidence rate was 26.0% for patients treated with 2D-RT, and 19.3% for all patients. For comparison, in a population-wide screening of 22,636 asymptomatic individuals^16^, the prevalence of carotid stenosis was 4.2%. A study of head and neck cancer, excluding symptomatic patients, found that the 4 year-incidence was 14%^14^. In another study, Lam *et al*. reported an incidence of 30% at 9 years after treatment in 71 cases^17^. Similar to many such studies published before, they included patients with cerebrovascular disease and carotid stenosis before RT^18^.

One study evaluated carotid stenosis of NPC patients by using contrast-enhanced MR angiography scanning. They found a rate of 37.5%, which is higher than our finding^19^. The difference may be attributed to different measurement methods as was discussed by the authors. Another study identified carotid stenosis (≥50%) in 20.9% of 129 NPC patients who underwent 2D-RT, similar with our result for all patients^20^. However, their published incidence rate was lower than ours for the 2D-RT group and higher for the IMRT group. Whether the difference is significant remains to be explored.

The present study illuminated that an older age might relate to a higher incidence of stenosis (≥50%) in NPC patients after RT treatment, consistent with the results of Zhou *et al*.^19^. They also found that the risk rose with time after RT. Post-radiation duration was judged to be the main cause of radiation-induced carotid stenosis. Previous studies evaluated intervals ranging from several months to over 20 years^21^. This indicates that the effect of irradiation was lasting; with the extension of time, the injurious effect on arteries would be more pronounced^8^. We found a difference of time interval in the significant stenosis group contrasted with the non-significant stenosis group, and this was statistically significant by multiple logistic regression analysis.

The hazard factors of stroke (smoking, diabetes, hypertension, vascular disease, atrial fibrillation, and carotid stenosis) have been widely demonstrated to give rise to carotid stenosis^22^. In our study, there was no significant difference between stenosis patients (≥50%) and non- stenosis patients (<50%). Even so, management of these risk factors is beneficial for NPC patients post-RT since it works for the general population^23^.

Our results suggest that IMRT significantly reduced the incidence of carotid stenosis after RT versus 2D-RT. Furthermore, CCA, ICA and carotid bulb in the IMRT group all showed a lower rate of significant stenosis than that in 2D-RT group. Lower incidence might be explained by the fact that the beams used by IMRT are composed of segments of different intensities^24^, which can provide more accurate dose distribution and save the surrounding tissues^25^.

Our results also suggest an RT dose effect for cerebrovascular events^26^. However, it is not feasible to eliminate dose to the carotids in clinical practice, even with IMRT, because the carotid arteries are often involved in the carotid nodal targeted volumes^27^.

No distinction was found for time interval from RT to the first color ultrasound duplex-detected significant carotid stenosis between patients treated with IMRT and those with 2D-RT. Interestingly, it has been reported that patients treated with 2D-RT had a longer time to develop radiation-induced temporal lobe injury compared with those treated with IMRT^9^. From our perspective, the time interval would be affected inevitably by the frequency and time of ultrasound examination. Since only a small proportion of patients with carotid stenosis will develop symptoms, most of them will not be diagnosed until the stenosis is detected by ultrasound or other examination during follow-up. Early detection of this complication can result in a better outcome. In our opinion, carotid color ultrasound should be a clinical routine during follow-ups for NPC patients after RT^28^.

The exact mechanism for carotid stenosis is not clear^29^. Injury to microvasculature may be the most important mechanism for injury in the large arteries^7^. Further research needs to be conducted in this area.

## Materials and Methods

### Patients

Our study reviewed all patients with NPC who underwent carotid ultrasound after 2D-RT or IMRT from 2006 to 2015 at Sun Yat-sen Memorial Hospital. This work was approved by the ethics committee of Sun Yat-sen Memorial Hospital and performed in accordance with the approved guidelines. All the participants provided informed consents. The exclusion criteria were history of transient ischemic attack (TIA), cerebrovascular disease, carotid stenosis, metastatic disease, and previous neck RT at diagnosis. No patients had NPC recurrence or a second primary cancer; those who were treated with palliative care, underwent carotid endarterectomy, or previous stenting were also excluded. The schedules of radiotherapy were collected from archived clinical records. Baseline demographic and comorbidity disease, including gender, age, smoking, and hypertension, diabetes, vascular disease, and concurrent chemotherapy were also included.

### Ultrasound technique

A carotid color-flow duplex scan was used with all patients. The study employed a color-coded duplex ultrasonograph with ATL HDI 3000 (Bothell, Wash), which combines a real-time B-mode image (5–10 MHz) that is used to acquire sagittal (anterior-posterior, posterior-anterior, lateral) and transverse views of the extracranial carotid system, with a pulsed-wave color Doppler flowmetry (3.0 MHz).

### Vascular stenosis diagnostic criteria

Carotid stenosis was diagnosed according to velocities (peak systolic, end diastolic) as well as artery ratios (internal carotid artery to common carotid artery). The reduction of 50% or greater in luminal diameter on either/both sides of the neck was regarded as significant stenosis based on previous publication^30^. The time interval was defined as interval from completion of RT to detection of carotid stenosis, or to the last ultrasound scan if no carotid stenosis was detected.

### Statistical analysis

Our data were expressed as mean ± SD (standard deviation) unless noted. Comparisons between groups were performed using t-test or x^2^ test. Multivariate logistic regression was adopted to estimate risk factors for carotid stenosis. SPSS 20.0 (SPSS Inc., IL, USA) was applied to perform statistical analysis. We considered *p* < 0.05 in two-tailed tests as significant.

## Conclusions

We believe that the present study was the first to make a comparison between IMRT and 2D-RT in terms of carotid stenosis in NPC patients. Our study revealed that older age was an independent hazard factor for significant carotid stenosis in irradiated NPC survivors, whereas IMRT was associated with decreased incidence of significant stenosis versus 2D-RT for NPC. Therefore, prevention and carotid ultrasound examination should be considered for older NPC survivors with longer interval from RT, especially those treated with 2D-RT.

## Data Availability

Due to ethical and legal restrictions, the data supporting findings presented in this manuscript are available from the corresponding author upon request.

## References

[1] Jemal A (2011). Global cancer statistics. CA Cancer J Clin.

[2] Wei WI, Sham JS (2005). Nasopharyngeal carcinoma. Lancet.

[3] Chua MLK, Wee JTS, Hui EP, Chan ATC (2016). Nasopharyngeal carcinoma. Lancet.

[4] Zhao Z (2013). Late-onset radiation-induced optic neuropathy after radiotherapy for nasopharyngeal carcinoma. J Clin Neurosci.

[5] Fang, W. *et al*. Late-onset cystic brain necrosis after radiotherapy for nasopharyngeal carcinoma. *Jpn J Clin Oncol*, 1–6, 10.1093/jjco/hyx028 (2017). 10.1093/jjco/hyx028 28334917

[6] Gu B (2014). Radiation-induced brachial plexus injury after radiotherapy for nasopharyngeal carcinoma. Jpn J Clin Oncol.

[7] Sano N (2015). Relationship between histologic features and outcomes of carotid revascularization for radiation-induced stenosis. Journal of vascular surgery.

[8] Xu J, Cao Y (2014). Radiation-induced carotid artery stenosis: a comprehensive review of the literature. Interventional neurology.

[9] Zhou GQ (2013). Radiation-induced temporal lobe injury for nasopharyngeal carcinoma: a comparison of intensity-modulated radiotherapy and conventional two-dimensional radiotherapy. PloS one.

[10] Yao JJ (2015). Incidence of and Risk Factors for Mastoiditis after Intensity Modulated Radiotherapy in Nasopharyngeal Carcinoma. PloS one.

[11] Yuan C, Wu VW, Yip SP, Kwong DL, Ying M (2017). Ultrasound Evaluation of Carotid Atherosclerosis in Post-Radiotherapy Nasopharyngeal Carcinoma Patients, Type 2 Diabetics, and Healthy Controls. Ultraschall in der Medizin (Stuttgart, Germany: 1980).

[12] Li CS, Schminke U, Tan TY (2010). Extracranial carotid artery disease in nasopharyngeal carcinoma patients with post-irradiation ischemic stroke. Clinical neurology and neurosurgery.

[13] Lee CC (2011). Increased risk of ischemic stroke in young nasopharyngeal carcinoma patients. International journal of radiation oncology, biology, physics.

[14] Dorth JA, Patel PR, Broadwater G, Brizel DM (2014). Incidence and risk factors of significant carotid artery stenosis in asymptomatic survivors of head and neck cancer after radiotherapy. Head & neck.

[15] Ou X, Xu T, He X, Ying H, Hu C (2017). Who benefited most from higher cumulative dose of cisplatin among patients with locally advanced nasopharyngeal carcinoma treated by intensity-modulated radiation therapy? A retrospective study of 527 cases. Journal of Cancer.

[16] de Weerd M, Greving JP, de Jong AW, Buskens E, Bots ML (2009). Prevalence of asymptomatic carotid artery stenosis according to age and sex: systematic review and metaregression analysis. Stroke.

[17] Lam WW (2001). Incidence of carotid stenosis in nasopharyngeal carcinoma patients after radiotherapy. Cancer.

[18] Stoker SD (2016). The Impact of the Overall Radiotherapy Time on Clinical Outcome of Patients with Nasopharyngeal Carcinoma; A Retrospective Study. PloS one.

[19] Zhou L (2015). Carotid and vertebral artery stenosis evaluated by contrast-enhanced MR angiography in nasopharyngeal carcinoma patients after radiotherapy: a prospective cohort study. The British journal of radiology.

[20] Yuan C, Wu VW, Yip SP, Kwong DL, Ying M (2014). Predictors of the extent of carotid atherosclerosis in patients treated with radiotherapy for nasopharyngeal carcinoma. PloS one.

[21] Simonetti G (2014). The role of radiotherapy in the carotid stenosis. Annali italiani di chirurgia.

[22] Mahmood SS, Levy D, Vasan RS, Wang TJ (2014). Lancet.

[23] Gujral DM (2014). Clinical features of radiation-induced carotid atherosclerosis. Clinical oncology (Royal College of Radiologists (Great Britain)).

[24] Kim YS (2016). Volumetric modulated arc therapy for carotid sparing in the management of early glottic cancer. Radiation oncology journal.

[25] Chen SW, Yang SN, Liang JA, Shiau AC, Lin FJ (2005). Comparative dosimetric study of two strategies of intensity-modulated radiotherapy in nasopharyngeal cancer. Med Dosim.

[26] Starke RM (2017). International multicenter cohort study of pediatric brain arteriovenous malformations. Part 2: Outcomes after stereotactic radiosurgery. Journal of neurosurgery. Pediatrics.

[27] Borghini A, Gianicolo EA, Picano E, Andreassi MG (2013). Ionizing radiation and atherosclerosis: current knowledge and future challenges. Atherosclerosis.

[28] Mahlmann A, Weiss N (2015). [Asymptomatic carotid artery stenosis–is screening useful?]. Deutsche medizinische Wochenschrift (1946).

[29] de Korte CL, Fekkes S, Nederveen AJ, Manniesing R, Hansen HR (2016). Review: Mechanical Characterization of Carotid Arteries and Atherosclerotic Plaques. IEEE transactions on ultrasonics, ferroelectrics, and frequency control.

[30] Nadareishvili ZG, Rothwell PM, Beletsky V, Pagniello A, Norris JW (2002). Long-term risk of stroke and other vascular events in patients with asymptomatic carotid artery stenosis. Arch Neurol.

